# Arhalofenate acid inhibits monosodium urate crystal-induced inflammatory responses through activation of AMP-activated protein kinase (AMPK) signaling

**DOI:** 10.1186/s13075-018-1699-4

**Published:** 2018-09-06

**Authors:** Charles McWherter, Yun-Jung Choi, Ramon L. Serrano, Sushil K. Mahata, Robert Terkeltaub, Ru Liu-Bryan

**Affiliations:** 1 CymaBay Therapeutics, Inc., Newark, California USA; 2 VA San Diego Healthcare System, 111K, 3350 La Jolla Village Drive, San Diego, CA 92161 USA; 3 University of California San Diego, La Jolla, California USA

**Keywords:** Gout, Inflammation, AMPK, Mitochondria, Autophagy flux

## Abstract

**Background:**

Arhalofenate acid, the active acid form of arhalofenate, is a non-agonist peroxisome proliferator-activated receptor γ (PPARγ) ligand, with uricosuric activity via URAT1 inhibition. Phase II studies revealed decreased acute arthritis flares in arhalofenate-treated gout compared with allopurinol alone. Hence, we investigated the anti-inflammatory effects and mechanisms of arhalofenate and its active acid form for responses to monosodium urate (MSU) crystals.

**Methods:**

We assessed in-vivo responses to MSU crystals in murine subcutaneous air pouches and in-vitro responses in murine bone marrow-derived macrophages (BMDMs) by enzyme-linked immunosorbent assay (ELISA), SDS-PAGE/Western blot, immunostaining, and transmission electron microscopy analyses.

**Results:**

Oral administration of arhalofenate (250 mg/kg) blunted total leukocyte ingress, neutrophil influx, and air pouch fluid interleukin (IL)-1β, IL-6, and CXCL1 in response to MSU crystal injection (*p* < 0.05 for each). Arhalofenate acid (100 μM) attenuated MSU crystal-induced IL-1β production in BMDMs via inhibition of NLRP3 inflammasome activation. In addition, arhalofenate acid dose-dependently increased activation (as assessed by phosphorylation) of AMP-activated protein kinase (AMPK). Studying AMPKα1 knockout mice, we elucidated that AMPK mediated the anti-inflammatory effects of arhalofenate acid. Moreover, arhalofenate acid attenuated the capacity of MSU crystals to suppress AMPK activity, regulated expression of multiple downstream AMPK targets that modulate mitochondrial function and oxidative stress, preserved intact mitochondrial cristae and volume density, and promoted anti-inflammatory autophagy flux in BMDMs.

**Conclusions:**

Arhalofenate acid is anti-inflammatory and acts via AMPK activation and its downstream signaling in macrophages. These effects likely contribute to a reduction of gout flares.

**Electronic supplementary material:**

The online version of this article (10.1186/s13075-018-1699-4) contains supplementary material, which is available to authorized users.

## Background

Anti-inflammatory prevention and treatment of attacks of gouty arthritis remain challenging, in part because many patients have incomplete responses or contraindications to one or more of the primary oral anti-inflammatory therapies (colchicine, nonsteroidal anti-inflammatory drugs (NSAIDs), and corticosteroids) [1, 2]. Moreover, gout flares often increase in frequency in the initial phase of urate-lowering therapy (ULT), thereby contributing to poor adherence to ULT and lack of improvement in health-related quality of life [1, 2]. Arhalofenate is a non-agonist ligand of peroxisome proliferator-activated receptor γ (PPARγ) with weak transactivation but robust transrepression activity [3]. It was first developed as an insulin sensitizer for type 2 diabetes mellitus [3]. Subsequently, arhalofenate was demonstrated to have uricosuric activity, as an inhibitor of URAT1, organic anion transporter 4 (OAT4) and OAT10 [4]. In a recent phase II trial in gout patients, which assessed acute gout flare as the primary endpoint, arhalofenate significantly reduced the risk of acute gouty arthritis in comparison with allopurinol alone, whereas there was no significant difference compared with allopurinol in combination with prophylactic colchicine [5]. The risk for urate-lowering therapy-induced gout flares depends on the degree of serum urate lowering [2]. Hence, this study was performed to directly test and characterize the anti-inflammatory effects of arhalofenate pertinent to gout.

Acute gouty arthritis is a characteristically severe phenotypic inflammatory response to deposits of monosodium urate (MSU) crystals which induce expression of NF-κB-dependent proinflammatory cytokines including pro-interleukin (IL)-1β and multiple chemokines [6, 7]. MSU crystals also stimulate activation of the NLRP3 inflammasome, with consequent maturation and release of IL-1β [6, 7]. This is a central driver of the gouty inflammation cascade which involves recruitment and activation of phagocytes [6, 7]. Core factors that modulate activation of the NLRP3 inflammasome, and experimental gout-like inflammation, include mitochondrial function, autophagy, and AMP-activated protein kinase (AMPK) [8, 9].

Mitochondrial reactive oxygen species (ROS) and oxidized mitochondrial DNA (mtDNA) promote inflammation [10–12], mediated by activation of NF-κB [10–12] and activation of the NLRP3 inflammasome via dysregulated balance between thioredoxins (TRXs) and thioredoxin-interacting protein (TXNIP) [13]. TRX1 and TRX2, mainly located in the cytoplasm and mitochondria, respectively, control cellular ROS by reduction of disulfides to thiol groups [14]. TXNIP directly binds to TRX and inhibits the reducing activity of TRX through disulfide exchange [14]. However, ROS triggers disassociation of TXNIP from TRX1, promoting direct physical interaction between TXNIP and NLRP3 that leads to activation of caspase-1 and release of mature IL-1β [13].

Autophagy mediates cellular homeostasis by degrading damaged proteins and organelles, including mitochondria [15–17]. Although MSU crystals promote autophagosome formation, the crystals also induce impairment of proteasomal degradation leading to accumulation of p62 [17]. As a selective autophagy receptor adaptor protein [17], p62 interacts with LC3-II to facilitate autophagic degradation [17], and also is involved in MSU crystal-induced caspase-1 activation and IL-1β release [18]. One of the major factors promoting autophagy is serine/threonine kinase AMPK [19].

AMPK is a nutritional biosensor that maintains cellular energy balance [19, 20], but nutritional excesses and other factors, including stimulation by MSU crystals and IL-1β, decrease AMPK activity [9]. Significantly, AMPK functions as an NF-κB and NLRP3 inflammasome inhibitor and promotes anti-inflammatory macrophage polarization, and markedly decreases the inflammatory response to MSU crystals in cultured macrophages [21, 22]. Moreover, AMPK transduces colchicine anti-inflammatory effects in vitro [22]. Pharmacologic AMPK activation markedly limits experimental gouty inflammation in the mouse in vivo using the subcutaneous air pouch model [22]. Conversely, MSU crystal-induced inflammation is prominently potentiated in AMPKα1 knockout (KO) mice [22].

Thiazolidinedione PPARγ agonists have been shown to cause phosphorylation and activation of AMPK [23–25]. Downstream targets of activated AMPK result in anti-inflammatory and cellular stress resistance effects that include PPARγ co-activator 1α (PGC-1α), the latter being a master regulator of mitochondrial biogenesis [26], as well as sirtuin 1 (SIRT1), which is a nicotinamide adenine dinucleotide (NAD)-dependent deacetylase [20, 26]. AMPK stimulates SIRT1 activity and phosphorylates the PGC-1α protein, which allows SIRT1 to deacetylate and activate PGC-1α [20, 26]. Activation of PGC-1α not only increases mitochondrial biogenesis by promoting expression of mitochondrial transcription factor A (TFAM), but also increases mitochondrial antioxidant capacity by upregulating expression of antioxidant enzymes [20, 26]. Here, we characterized anti-inflammatory effects of arhalofenate in vivo and the in-vitro mechanisms of action of arhalofenate acid (the circulating active acid form of arhalofenate) in MSU crystal-induced macrophage activation. Our results implicate AMPK through its downstream signaling actions in impacting mitochondria, TRX and TXNIP, and autophagy as a central mediator of the anti-inflammatory activity of arhalofenate.

## Methods

### Reagents

All chemical reagents were from Sigma-Aldrich (St. Louis, MO) unless otherwise stated. Arhalofenate acid (MBX-102 acid), the active form of arhalofenate, was used for in-vitro studies. Arhalofenate (MBX-102) was used for in-vivo studies. MSU crystals were prepared as described previously [22], suspended at 25 mg/mL in sterile, endotoxin-free phosphate-buffered saline (PBS), and verified to be free of detectable lipopolysaccharide contamination by Limulus lysate assay (Lonza, Walkersville, MD). A-769662 was from LC laboratories (Woburn, MA). Antibodies to phospho-AMPKα (Thr172) and total AMPKα (recognizing both AMPKα isoforms), SIRT1, TFAM, TRX1, TRX2, and TXNIP were from Cell Signaling Technology (Danvers, MA). Antibodies to pro-caspase-1 and cleaved caspase-1 (p10) were from Biovision (Milpitas, CA) and Santa Cruz Biotechnology (Santa Cruz, CA), respectively.

### Subcutaneous air pouch model and flow cytometry analysis

C57BL/6 mice (*n* = 8–10/group) were subcutaneously injected under the skin adjacent to the back of the neck with sterile air (day 1, 5 mL; day 4, 3 mL) to form air pouches as described previously [22]. On day 4, mice were dosed daily with vehicle (1% carboxymethylcellulose/2% Tween-80), arhalofenate (at a loading dose of 250 mg/kg per oral), or dexamethasone (20 mg/kg intraperitoneally) for 3 days. On day 7, 30 min after the last dose, MSU crystals (20 mg in 5 mL saline) were injected into the air pouch to elicit an acute immune response. After 4 h, the mice were sacrificed and 5 mL of heparinized saline was injected into the air pouch to collect the exudates.

Exudates were centrifuged and resuspended in PBS. The cell suspension was incubated with phycoerythrin (PE)-conjugated rat anti-mouse CD45^+^ antibody (diluted 1:100; MCA1031PE; AbD Serotech) for all leucocyte staining, and fluorescein isothiocyanate (FITC)-conjugated rat anti-mouse Ly-6B.2 alloantigen (diluted 1:100; #MCA771FB; AbD Serotech) for neutrophil staining according to the manufacturer’s protocol. Propidium iodide staining solution (BD Pharmingen™; #556463) was added to the cell suspension to exclude non-viable cells. All live cells were further gated based on CD45 expression for leukocytes. BD™ Compbeads (BD Biosciences; #552845) were used for nonspecific binding of antibodies to optimize fluorescence compensation settings. Counting beads (Spherotech Accuount fluorescent particles; #ACFP-100-3) were added to the stained cells to obtain absolute cell number. FACS analysis was performed on a BD LSR II flow cytometer using Diva software (v6.1.2, Becton Dickinson) and analyzed using FlowJo software (v9.5.3, Tree Star Inc.).

### Cell culture

Bone marrow-derived macrophages (BMDMs) were generated as described previously [22]. Briefly, bone marrow cells were cultured in complete RPMI media containing 10% fetal bovine serum (FBS), penicillin (100 U/mL), and streptomycin (100 μg/mL) in the presence of macrophage colony-stimulating factor (M-CSF; 20 ng/mL; Gemini Bio-products, West Sacramento, CA). After 5–7 days, the M-CSF-derived macrophages were re-plated onto 24-well (5 × 10^5^/well) or six-well (2 × 10^6^/well) plates and primed with 20 ng/mL granulocyte-macrophage colony-stimulating factor (GM-CSF; Gemini Bio-products, West Sacramento, CA) for 24 h in complete RPMI medium before treatment with the indicated reagents in fresh RPMI containing only 1% FBS.

### Western blot

Cells were lysed in RIPA buffer with 2 mM sodium vanadate and protease inhibitor cocktails (Roche, Mannheim, Germany). Cell lysates (10–15 μg) were separated by gradient 4–20% SDS-PAGE and transferred onto nitrocellulose membranes (Bio-Rad, Hercules, CA), probed with antibodies, exposed to SuperSignal West Pico Chemiluminescent Substrate (Thermo Scientific, Waltham, MA), and visualized by radiography.

### Cytokine analyses

Mouse IL-1β and CXCL1 (KC) were measured using DuoSet enzyme-linked immunosorbent assay (ELISA; R&D Systems, Minneapolis, MN).

### Fluorescence microscopy

BMDMs were incubated with MitoSOX Red reagent (Thermo Scientific) reagent (1 μM) to examine mitochondrial ROS generation which was visualized by fluorescence microscopy. Cells were also incubated with 10 nM MitoTracker Green (which is insensitive to ROS) to confirm the localization of MitoSOX Red to mitochondria. Immunofluorescence microscopy was carried out to visually identify p62 puncta and lysosomes and to determine co-localization of p62 and lysosomal-associated membrane protein 1 (LAMP1), which indicates autophagosome and lysosome fusion (i.e., activated autophagy). In brief, cells were fixed and permeabilized with cold methanol. Immunocytochemical staining of cells used rabbit anti-p62 monoclonal antibody (Cell Signaling, #23214) or rabbit anti-LAMP1 antibody (Abcam, #ab24210). Alexa Fluor 488 goat anti-rabbit IgG (Thermo Scientific) and Alexa Flour 555 goat anti-rabbit IgG (Thermo Scientific) secondary antibodies were used to detect p62 and LAMP1, respectively. Imaging was acquired via a confocal microscope (Zeiss LSM 880 Confocal with FAST Airyscan).

### Transmission electron microscopy (TEM)

Cells were fixed with 2.5% glutaraldehyde in 0.15 M cacodylate buffer, and postfixed in 1% OsO_4_ in 0.1 M cacodylate buffer for 1 h on ice, followed by staining en bloc with 2–3% uranyl acetate for 1 h on ice. The cells were dehydrated in a graded series of washes with ethanol (20–100%) on ice followed by one wash with 100% ethanol and two washes with acetone (15 min each) and embedded with Durcupan. Ultrathin (50–60 nm) sections were cut on a Leica UCT ultramicrotome, and picked up on Formvar and carbon-coated copper grids. Sections were stained with 2% uranyl acetate for 5 min and Sato’s lead stain for 1 min. Grids were viewed using a JEOL JEM1400-plus TEM (JEOL, Peabody, MA). TEM images were taken using a Gatan OneView digital camera with 4 k × 4 k resolution (Gatan, Pleasanton, CA). Mitochondrial area was determined using the free-hand tool in ImageJ and manually tracing around the mitochondrial outer membrane. The area of each crista membrane was also calculated in the same manner. The sum of the areas of the total complement of cristae was then divided by the sum of the mitochondrial area to obtain the cristae volume density as described previously [27].

### Statistical analyses

Data are presented as either mean values ± standard deviation (SD) or mean ± standard error of the mean (SEM) as indicated. Statistical analyses were performed by one-way or two-way analysis of variance with Bonferroni post-hoc testing using GraphPad Prism software, version 6. *p* values less than 0.05 were considered significant.

## Results

### Arhalofenate attenuated MSU crystal-induced inflammation in mice in vivo

**Fig. 1:**
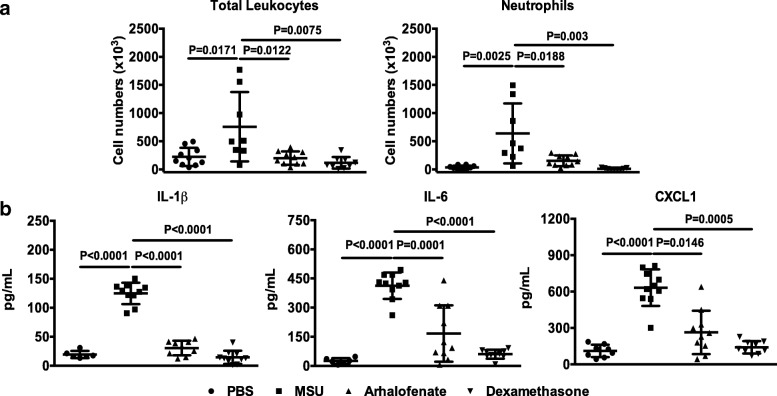
Arhalofenate attenuates MSU crystal-induced inflammation in mice in vivo. Air pouches were created in normal C57BL/6 mice, and mice were subsequently dosed with arhalofenate orally for 3 days prior to the introduction of monosodium urate (MSU) crystals into the air pouch as described in the Methods section. The acute inflammatory response to crystal injection was determined by measuring the number of total infiltrating leukocytes or neutrophils (**a**), and production of interleukin (IL)-1β, IL-6 and CXCL1 (**b**) in the air pouch exudate 4 h post-dose. Dexamethasone served as the anti-inflammatory control agent. Data are shown as the mean ± SEM (*n* = 8–10 mice per group). The *p* values represent comparisons between MSU crystals alone and the phosphate-buffered saline (PBS) control, or between MSU crystals alone and MSU crystals plus arhalofenate or dexamethasone

In the murine subcutaneous air pouch model of acute gouty inflammation, arhalofenate significantly inhibited leukocyte or neutrophil infiltration and production of IL-1β, IL-6, and CXCL1 induced by MSU crystals (Fig. 1). The effects of arhalofenate in this model were comparable with those of the positive control dexamethasone, and indicated the capacity of arhalofenate to limit MSU crystal-induced inflammation. Of note, arhalofenate or dexamethasone alone did not exhibit any toxicity effect (data not shown).

### Arhalofenate acid suppressed MSU crystal-induced NLRP3 inflammasome activation

**Fig. 2:**
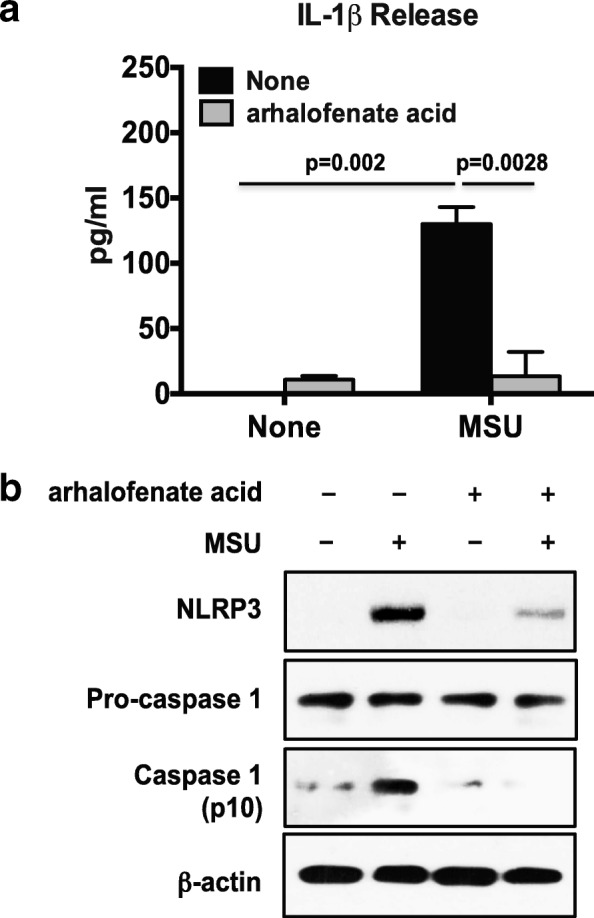
Arhalofenate acid attenuates MSU crystal-induced IL-1β release by inhibiting NLRP3 inflammasome activation in BMDMs in vitro. BMDMs were pretreated with arhalofenate acid at a concentration of 100 μM for 1 h before being stimulated with monosodium urate (MSU) crystals (0.2 mg/mL) in RPMI containing 1% FBS for 18 h. The conditioned media was used for ELISA for interleukin (IL)-1β (**a**), and the cell lysates were subjected to Western blot analysis (**b**) for expression of NLRP3, pro-caspase 1, and cleaved caspase 1 (p10). Data in **a** are the mean ± SD of three individual experiments, and *p* values represent comparisons between none and MSU crystals alone, or between MSU crystal alone and MSU crystals plus arhalofenate acid. Data in **b** are representative of three individual experiments

Arhalofenate acid (100 μM) inhibited MSU crystal-induced IL-1β release in cultured murine BMDMs (Fig. 2a). Partial inhibition of MSU crystal-induced IL-1β release was observed with lower doses at 25 and 50 μM (data not shown). No cytotoxicity of arhalofenate at the concentrations studied was observed (data not shown). Western blot analysis showed that the level of NLRP3 protein expression increased in response to MSU crystals, an effect reduced by arhalofenate acid (Fig. 2b). Similarly, MSU crystal-induced expression of cleaved caspase-1 was diminished by arhalofenate acid, indicating inhibition of MSU crystal-induced NLRP3 inflammasome activation (Fig. 2b).

### Arhalofenate acid induced functionally important AMPK activation

**Fig. 3:**
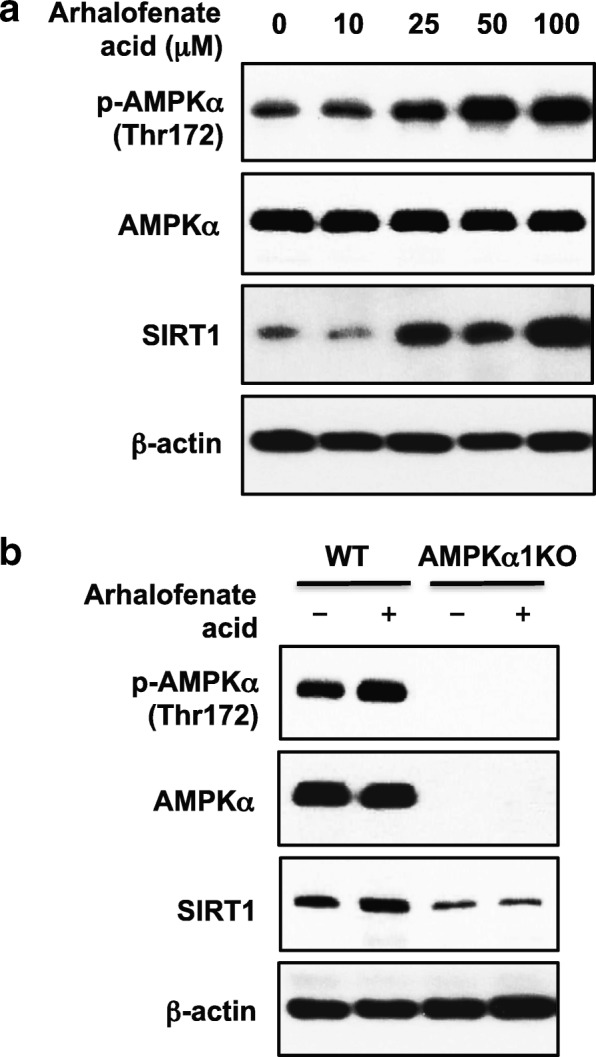
Arhalofenate acid induced phosphorylation of AMPKα and expression of SIRT1 in BMDMs in vitro. BMDMs prepared from wild-type (WT) and AMPKα1 knockout (KO) mice were pretreated with arhalofenate acid at the concentrations indicated for 18 h (**a**) or at 100 μM for 1 h (**b**) in RPMI containing 1% FBS. Cell lysates were subjected to Western blot analysis for phosphorylation (p) and expression of AMP-activated protein kinase (AMPK)α and sirtuin 1 (SIRT1). Data in both **a** and **b** are representative of three individual experiments

**Fig. 4:**
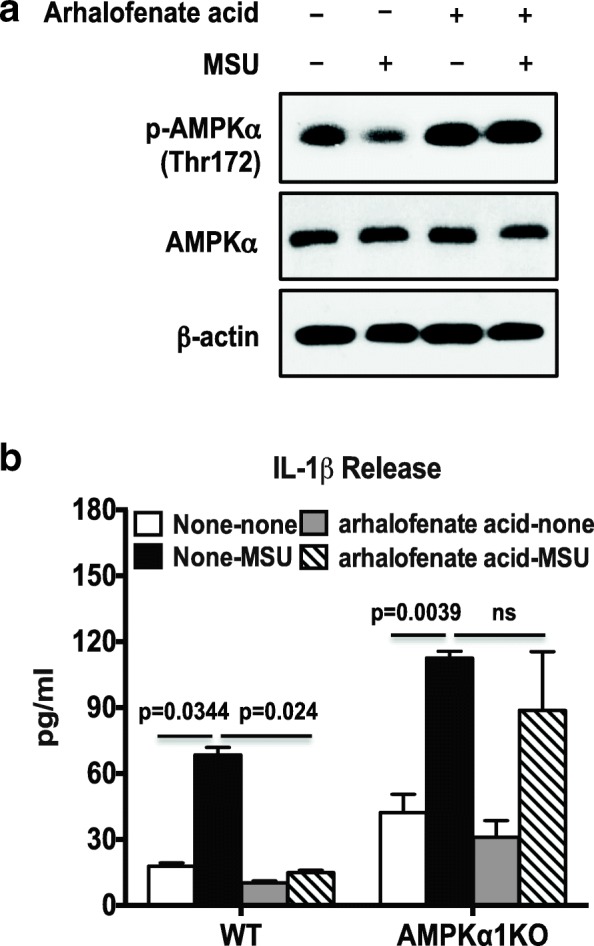
Arhalofenate acid inhibited MSU crystal-induced IL-1β via AMPK in BMDMs in vitro. BMDMs were treated with arhalofenate acid at 100 μM for 1 h before being stimulated with monosodium urate (MSU) crystals (0.2 mg/mL) for 18 h in RPMI containing 1% FBS. Cell lysates were subjected to Western blot analysis for phosphorylation (p) and expression of AMP-activated protein kinase (AMPK)α (**a**). The conditioned medium was used for ELISA analysis of interleukin (IL)-1β release (**b**). Data in **a** are representative of three individual experiments. Data in **b** are the mean ± SD of three individual experiments. The *p* values in **b** represent comparisons between none and MSU crystals alone in the presence or absence of arhalofenate acid in either wild-type (WT) or AMPKα1 knockout (KO) BMDMs. ns not significant

Arhalofenate acid enhanced phosphorylation of AMPKα and expression of SIRT1 in a dose-dependent manner (Fig. 3a). Notably, the basal level of SIRT1 expression was reduced in AMPKα1KO BMDMs, and arhalofenate acid failed to enhance SIRT1 expression in AMPKα1KO BMDMs (Fig. 3b). These results indicated that SIRT1 was a downstream target of AMPK, and that arhalofenate acid-induced increase in SIRT1 expression was dependent on AMPK. Arhalofenate acid inhibited the capacity of MSU crystals to reduce AMPK activity (Fig. 4a), and correlated with suppressed IL-1β release (Fig. 4b) in wild-type BMDMs. However, arhalofenate acid failed to significantly limit IL-1β release in AMPKα1KO BMDMs (Fig. 4b).

### Arhalofenate acid modulated AMPK-regulated downstream mitochondrial targets and ultrastructure

**Fig. 5:**
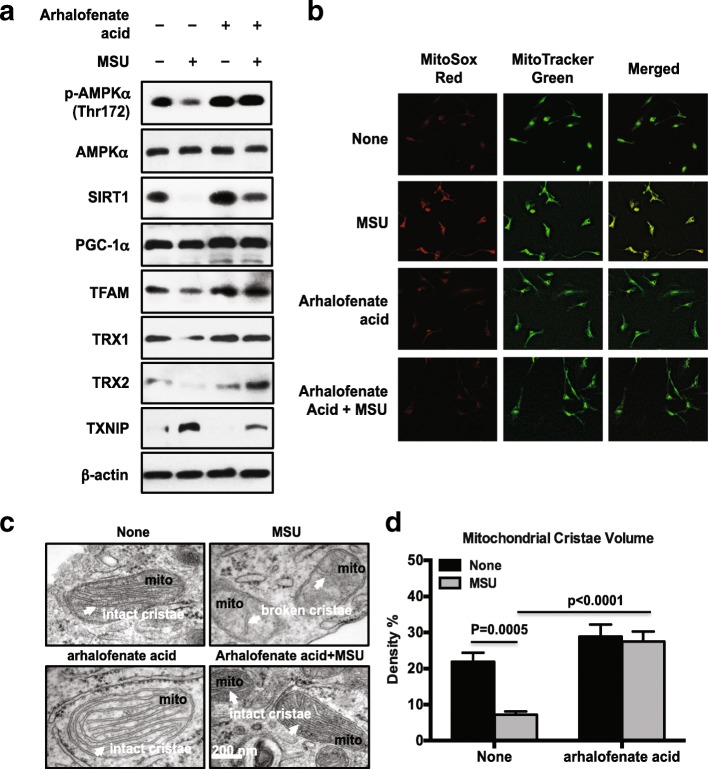
Arhalofenate acid activated AMPK downstream targets involved in regulation of mitochondrial function and maintained mitochondrial cristae area. BMDMs were treated with arhalofenate acid (100 μM) for 1 h before stimulation with monosodium urate (MSU) crystals (0.2 mg/mL) for 1 h or 18 h in RPMI containing 1% FBS. Cell lysates prepared from 18-h treatment samples were subjected to Western blot analysis of phosphorylation (p) and expression of AMP-activated protein kinase (AMPK)α, expression of sirtuin 1 (SIRT1), peroxisome proliferator-activated receptor γ co-activator 1α (PGC-1α), and mitochondrial transcription factor A (TFAM), and expression of thioredoxin (TRX)1, TRX2, and thioredoxin-interacting protein (TXNIP) (**a**). Cells from 1-h treatment were then stained with MitoSOX Red and MitoTracker Green and visualized by fluorescence microscopy (**b**, magnification 20×). TEM analysis was performed to examine mitochondrial cristae (the folds of inner mitochondrial membrane indicated by white arrows in **c**), and the cristae volume density are presented (**d**). Data in **a** and **b** are representative of three individual experiments. Data in **c** are representative of 30 cells examined for each condition. Data in **d** are the mean ± SEM of 30 cells. The *p* values represent comparisons between none and MSU crystals alone, or between MSU crystals alone and MSU crystals plus arhalofenate acid

MSU crystals concurrently reduced AMPK activity (assessed by phosphorylation of AMPKα) and the expression of SIRT1, PGC-1α, and TFAM, with all of these effects limited by arhalofenate acid (Fig. 5a). In addition, arhalofenate acid also enhanced basal levels of SIRT1, PGC-1α, and TFAM, with similar results seen with treatment with the selective AMPK activator A-769662 (Additional file 1). Moreover, MSU crystals induced mitochondrial ROS generation, evidenced by prominent MitoSOX Red staining (Fig. 5b). MitoTracker Green staining confirmed the localization of MitoSOX Red to mitochondria (Fig. 5b). Notably, arhalofenate acid was able to inhibit this effect (Fig. 5b). TEM studies revealed that macrophages stimulated with MSU crystals exhibited broken cristae with a consequent decrease in cristae volume density. Interestingly, arhalofenate prevented MSU crystal-induced loss of intact mitochondrial cristae, the folds in the inner membrane of mitochondria that provide the high surface area for oxidative phosphorylation (OXPHOS) for generation of ATP (Fig. 5c). The protective effects of arhalofenate acid on mitochondrial respiratory function were associated with preservation of mitochondrial ultrastructure, supported by the ability of arhalofenate acid to maintain cristae volume density (Fig. 5d). Since AMPK activation has been shown to inhibit TXNIP expression [28, 29], we next assessed the effects of arhalofenate acid on expression of TRX and TXNIP. As seen in Fig. 5a, MSU crystals decreased the expression of TRX1 and TRX2 isoforms, and induced the expression of TXNIP. These effects were inhibited by arhalofenate acid, suggesting an ability of arhalofenate acid for maintaining the balance between the TRX isoforms and TXNIP. Interestingly, the levels of TRX2 expression were noticeably higher in cells treated with MSU crystals in the presence of arhalofenate acid compared with cells treated with arhalofenate acid alone.

### Arhalofenate acid prevented MSU crystal-induced p62 accumulation by promoting autophagy flux

**Fig. 6:**
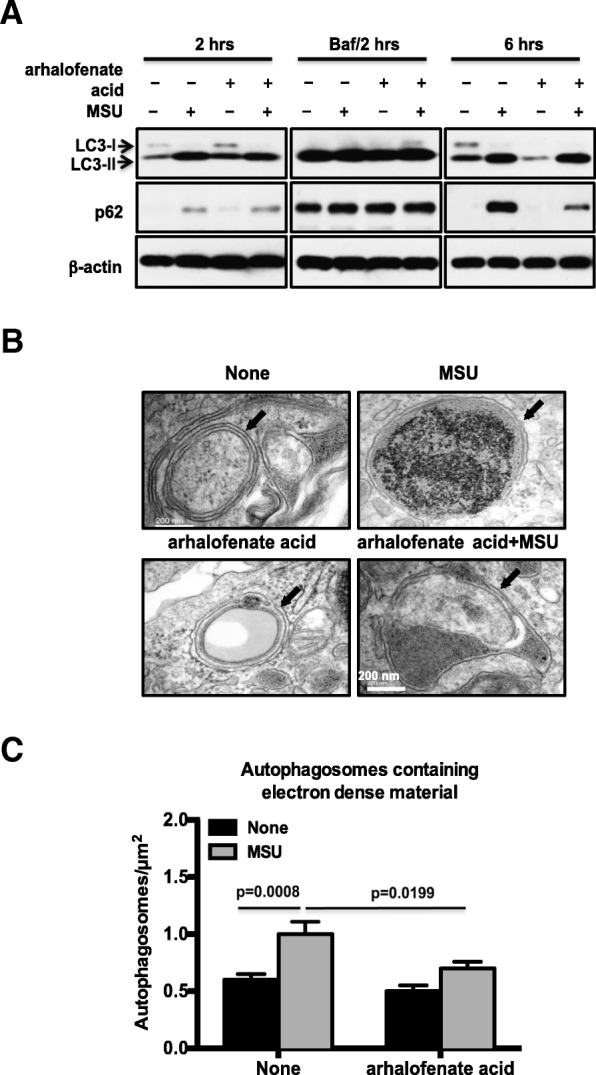
Arhalofenate acid prevented prolonged accumulation of p62 by promoting autophagy flux in response to MSU crystals. BMDMs were treated with arhalofenate acid (100 μM) for 1 h before stimulated with monosodium urate (MSU) crystals (0.2 mg/mL) for 2 h in the presence or absence of bafilomycin (Baf; 100 nM) and for 6 h in RPMI containing 1% FBS. Cell lysates were subjected to Western blot analysis of LC3 and p62 (**a**). TEM was performed to examine autophagosomes (indicated by black arrows in **b**), and the numbers of autophagosomes containing electron dense material per μm^2^ are presented (**c**). Data in **a** and **b** are representative of three individual experiments. Data in **c** are the mean ± SEM of 30 cells. The *p* values represent comparisons between none and MSU crystals alone, or between MSU crystals alone and MSU crystals plus arhalofenate acid

We first confirmed [18] that MSU crystals increased the expression of LC3-II, the lipidated form of LC3, indicating autophagosome formation at both 2 and 6 h in BMDMs (Fig. 6a). Expression of p62 was also induced at 2 h and greatly enhanced at 6 h by MSU crystals. Bafilomycin, a known inhibitor of the late phase of autophagy that prohibits phagosome and lysosome fusion and thereby inhibits autophagy flux, caused accumulation of LC3-II and p62 regardless of the presence or absence of MSU crystals and arhalofenate acid. Notably, arhalofenate acid had a minimal effect on MSU-induced expression of LC3-II at 2 and 6 h, and markedly decreased MSU crystal-induced p62 expression at 6 h, but not at 2 h (Fig. 6a). A similar result was observed with A-769662 (Additional file 1). Immunofluorescence analysis of p62 (green color) and LAMP1 (red color) showed significantly less yellow punctae (co-localization of p62 and LAMP1) in MSU crystal-treated cells in the presence of arhalofenate acid in comparison with the cells treated with MSU crystals alone (Additional file 2). TEM analysis demonstrated that cells treated with MSU crystals alone at 6 h had large amounts of electron dense material within the phagosomes, which were considerably less abundant when arhalofenate acid was also present (Fig. 6b). In addition, the number of autophagosomes containing dense debris was significantly smaller under the same conditions with arhalofenate acid treatment (Fig. 6c). Collectively, the results pointed to the capacity of arhalofenate acid to promote autophagy flux, thereby preventing accumulation of p62 induced by MSU crystals.

## Discussion

This study demonstrated that the uricosuric arhalofenate (and its active acid form) has anti-inflammatory effects, and it also characterized the molecular mechanism of action for these activities of MSU crystal-induced inflammation. We discovered that arhalofenate acid inhibited MSU crystal-induced inflammatory responses through activation of AMPK and AMPK downstream signaling. This enabled cellular resistance to stresses induced by MSU crystals, largely by maintaining mitochondrial function and cellular quality control through autophagy.

We confirmed that arhalofenate attenuated MSU crystal-induced inflammatory responses in a murine air pouch model in vivo, and that arhalofenate acid mitigated MSU crystal-induced IL-1β production by inhibiting NLRP3 inflammasome activation in macrophages in vitro. Moreover, we found that arhalofenate acid, which is a non-agonist ligand of PPARγ, induced phosphorylation of AMPKα in a dose-dependent manner in macrophages. It is known that PPARγ agonists (e.g., thiazolidinedione drugs) can activate AMPK by phosphorylation of AMPKα [30]. Furthermore, we have previously demonstrated that activation of AMPK attenuated MSU crystal-induced inflammatory responses through inhibition of NLRP3 inflammasome activation [22]. The hypothesis that arhalofenate acid was acting in an AMPK-dependent manner to exert anti-inflammatory effects was strongly supported by the data that arhalofenate acid was no longer able to significantly inhibit MSU crystal-induced IL-1β release in macrophages deficient in AMPKα1, the predominant α isoform of AMPK in macrophages.

In this study, arhalofenate acid, and similarly the AMPK selective activator A-769662, prevented MSU crystal-induced decrease in phosphorylation of AMPKα, and the expression of SIRT1, PGC-1α, and TFAM. In addition, we found that arhalofenate acid prohibited the loss of mitochondrial cristae induced by MSU crystals. As such, arhalofenate acid was demonstrated to have the ability to regulate mitochondrial function not only via AMPK and downstream signaling, but also by preserving mitochondrial ultrastructure. The significance of our findings stems partly from the recent emergence of mitochondria as central regulators of NLRP3 inflammasome activation [10–12]. The NLRP3 inflammasome can sense danger-associated signals that are induced by defective mitochondria including mitochondrial ROS and oxidized mtDNA [10–12]. The major function of mitochondria is to generate ATP through the process of OXPHOS. Defects in the electron transport chain (the transport system that generates the major amount of ATP through OXPHOS) are usually detrimental to the host. Our TEM analysis clearly demonstrated that MSU crystals induced the breakdown of mitochondrial cristae in macrophages, with a consequent decrease in cristae volume density, and that the cristae are known to provide a large surface area in the inner membrane of the mitochondria for OXPHOS to generate ATP. Inhibition of mitochondrial complex-I by rotenone or complex-III by antimycin A induces robust ROS production by mitochondria [31, 32]. This enhanced ROS production is sufficient to drive NLRP3 inflammasome activation [10–12]. The oxidized mtDNA released from damaged mitochondria directly binds NLRP3 to activate the inflammasome [10–12]. Activation of AMPK is known to promote the oxidative metabolism and mitochondrial biogenesis via the downstream targets SIRT1 and PGC-1α [20]. Activation of PGC-1α leads to increased expression of the mitochondrial transcription factor TFAM, which in turn stimulates mitochondrial DNA replication [26].

Increased TRX1 and decreased TXNIP are beneficial for preventing hyperinflammation, neurodegeneration, and the progression of diabetes [14]. In this study, we found that arhalofenate acid, similar to the effects of A-769662, increased basal levels of expression of TRX1 and TRX2 and restrained MSU crystals from reducing the expression of TRX1 and TRX2 and inhibited MSU crystal-induced TXNIP expression. We observed that the levels of TRX2 expression were even higher in cells treated with MSU crystals in the presence of arhalofenate acid compared with cells treated with arhalofenate acid alone. The mechanism responsible for this paradoxically higher TRX2 expression in response to the combination of MSU crystals and arhalofenate remains to be determined and was beyond the scope of this study. TXNIP has been shown to translocate to the mitochondria where it binds to oxidized TRX2 leading to increasing ROS accumulation and mitochondrial dysfunction [14]. TRX2 binds to apoptosis signaling regulating kinase 1 (Ask1). Increasing binding of TXNIP to TRX2 reduces the interaction between TRX2 and Ask1, and induces Ask1 activation for apoptosis [14]. Although dysregulation of the balance between TRX1 and TXNIP is involved in NLRP3 inflammasome activation in a redox-dependent manner [13], the functions of TRX2 and TXNIP in regulating the NLRP3 inflammasome are unclear. We speculate that arhalofenate acid regulates the expression of TRX and TXNIP through AMPK signaling. In this context, metformin increases TRX expression through activation of AMPK [33], and it also inhibits TXNIP expression [34]. Moreover, AMPK mediates nutrient regulation of TXNIP expression [28].

In this study, we showed that arhalofenate acid prevented the prolonged accumulation of p62 induced by MSU crystals, indicating that arhalofenate acid may inhibit MSU crystal-induced NLRP3 inflammasome activation partly by improving autophagy flux. Autophagy is a fundamental cellular process that is required for clearance of damaged and dysfunctional organelles, such as mitochondria [15–17]. Incomplete clearance of damaged mitochondria can trigger aberrant inflammasome activation and promote a variety of human inflammatory diseases [15–17]. In macrophages, autophagy blockade increases the production of mitochondrial ROS that induces mitochondrial damage, in turn activating the inflammasome [12]. Autophagy consists of four essential steps: initiation of autophagosome formation, elongation and closure of autophagic membrane, fusion between autophagosome and lysosome, and degradation [35]. MSU crystals induce autophagosome formation and lipidation of LC3 (conversion from LC3-I to LC3-II) [18]. However, MSU crystals also induce accumulation of p62, a selective autophagy adaptor for degradation of ubiquitinated substrates [18]. Since p62 has an LC3-binding motif, p62 binds with LC3 on the autophagosome and facilitates autophagic degradation [36]. Levels of p62 usually inversely correlate with autophagic degradation in later stages of autophagy [36]. Although studies have shown that p62 is increased and translocated to damaged mitochondria in NLRP3 inflammasome-activated cells, the detailed molecular mechanism on the link between the NLRP3 inflammasome and autophagy, especially mitophagy, is not yet fully understood. Interestingly, recent studies reported that, on stimulation of macrophages with NLRP3 inflammasome activators, p62, whose expression is induced via NF-κB, LC3-II, and Parkin, were recruited to the damaged mitochondria, initiating organelle clearance via mitophagy [36, 37]. This “NF-κB-p62-mitophagy” signaling axis represents a key macrophage-intrinsic regulatory mechanism that keeps NLRP3 inflammasome activation in check [37, 38]. Further studies on how arhalofenate acid controls MSU crystal-induced NLRP3 inflammation related to mitophagy will be of interest.

## Conclusion

Arhalofenate acid, the active acid form of arhalofenate, exerts anti-inflammatory effects in MSU crystal-treated macrophages. These effects were mediated to a large degree by inducing AMPK activation, and at least in part by associated maintenance of mitochondrial function through activation of AMPK and its downstream signaling and preservation of mitochondrial cristae surface area, and by increasing cellular quality control by promoting autophagy. The results of this study identify basic mechanisms by which arhalofenate treatment decreases acute flares in patients with gout [5].

## Additional files

Additional file 1: A-769662 activated AMPK downstream targets involved in regulation of mitochondrial function. BMDMs were treated with direct AMPK activator A-769662 (100 μM) for 1 h before being stimulated with MSU crystals (0.2 mg/mL) for 6 or 18 h in RPMI containing 1% FBS. Western blot analysis was carried out to examine phosphorylation and expression of AMPKα, expression of SIRT1, PGC-1α, and TFAM, and expression of TXN1, TXN2, and TXNIP from 18-h treatment cells (A), and expression of LC3 and p62 from 6-h treatment cells (B). Data shown in A and B are representative of three individual experiments. (PDF 753 kb)

Additional file 2: Arhalofenate acid promoted autophagy flux. BMDMs were treated with arhalofenate acid (100 μM) for 1 h before being stimulated with MSU crystals (0.2 mg/mL) for 6 h in RPMI containing 1% FBS. Immunofluorescence microscopy was carried out to visually identify p62 puncta (green) and lysosomes (LAMP1, red), and determine co-localization (yellow) of p62 and LAMP1 (A, 63×). The numbers of yellow punctae per cell were counted and presented in a graph (B). Data in A are representative of three individual experiments. Data in B are the mean ± SD of 200 cells examined for each condition. The *p* values represent comparisons between none and MSU crystals alone, or between MSU crystals alone and MSU crystals plus arhalofenate acid. (PDF 38585 kb)

## Abbreviations

AMPK: AMP-activated protein kinase
Ask1: Apoptosis signaling regulating kinase 1
BMDM: Bone marrow-derived macrophage
FBS: Fetal bovine serum
GM-CSF: Granulocyte-macrophage colony-stimulating factor
IL: Interleukin
KO: Knockout
LAMP1: Lysosomal-associated membrane protein 1
M-CSF: Macrophage colony-stimulating factor
MSU: Monosodium urate
mtDNA: Mitochondrial DNA
NAD: Nicotinamide adenine dinucleotide
OXPHOS: Oxidative phosphorylation
PBS: Phosphate-buffered saline
PGC-1α: Peroxisome proliferator-activated receptor γ co-activator 1α
PPARγ: Peroxisome proliferator-activated receptor γ
ROS: Reactive oxygen species
SIRT1: Sirtuin 1
TEM: Transmission electron microscopy
TFAM: Mitochondrial transcription factor A
TRX: Thioredoxin
TXNIP: Thioredoxin-interacting protein
