# Supplementary material for: Arhalofenate acid inhibits monosodium urate crystal-induced inflammatory responses through activation of AMP-activated protein kinase (AMPK) signaling
Source: Arthritis Res Ther. 2018 Sep 6;20:204. doi: 10.1186/s13075-018-1699-4 (PMC6127987; doi:10.1186/s13075-018-1699-4)
Supplement: Supplementary file 1 — A-769662 activated AMPK downstream targets involved in regulation of mitochondrial function. BMDMs were treated with direct AMPK activator A-769662 (100 μM) for 1 h before being stimulated with MSU crystals (0.2 mg/mL) for 6 or 18 h in RPMI containing 1% FBS. Western blot analysis was carried out to examine phosphorylation and expression of AMPKα, expression of SIRT1, PGC-1α, and TFAM, and expression of TXN1, TXN2, and TXNIP from 18-h treatment cells (A), and expression of LC3 and p62 from 6-h treatment cells (B). Data shown in A and B are representative of three individual experiments. (PDF 753 kb) [file 13075_2018_1699_MOESM1_ESM.pdf]

Additional file 1

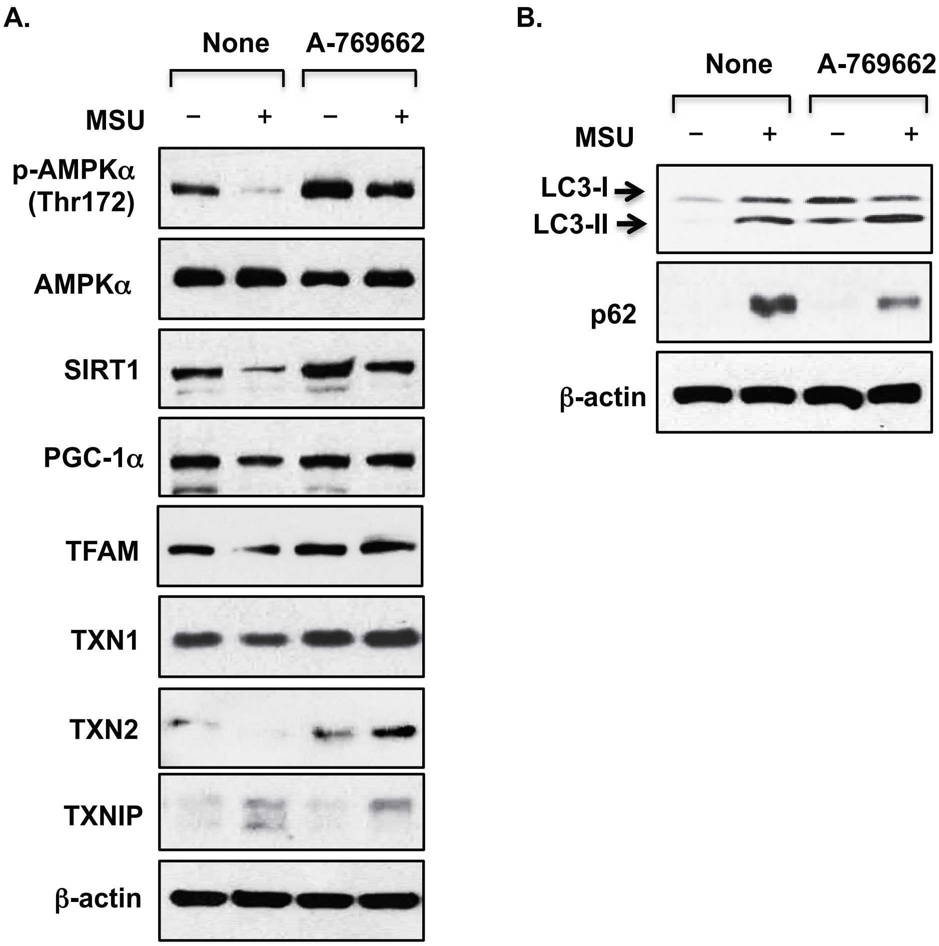

Additiona File 1. A-769662 activated AMPK downstream targets involved in regulation of mitochondrial function. BMDMs were treated with direct AMPK activator A-769662 (100 μM) for 1 hour before stimulated with MSU crystals (0.2 mg/ml) for 6 or 18 hours in RPMI containing 1% FBS. Western blot analysis was carried out to examine phosphorylation and expression of AMPKα, expression of SIRT1, PGC-1α and TFAM, and expression of TXN1, TXN2 and TXNIP from 18 hours treatment cells (A), and expression of LC3 and p62 from 6 hours treatment cells (B). Data shown in A and B as representative of 3 individual experiments.
