# Supplementary material for: Arhalofenate acid inhibits monosodium urate crystal-induced inflammatory responses through activation of AMP-activated protein kinase (AMPK) signaling
Source: Arthritis Res Ther. 2018 Sep 6;20:204. doi: 10.1186/s13075-018-1699-4 (PMC6127987; doi:10.1186/s13075-018-1699-4)
Supplement: Supplementary file 2 — Arhalofenate acid promoted autophagy flux. BMDMs were treated with arhalofenate acid (100 μM) for 1 h before being stimulated with MSU crystals (0.2 mg/mL) for 6 h in RPMI containing 1% FBS. Immunofluorescence microscopy was carried out to visually identify p62 puncta (green) and lysosomes (LAMP1, red), and determine co-localization (yellow) of p62 and LAMP1 (A, 63×). The numbers of yellow punctae per cell were counted and presented in a graph (B). Data in A are representative of three individual experiments. Data in B are the mean ± SD of 200 cells examined for each condition. The p values represent comparisons between none and MSU crystals alone, or between MSU crystals alone and MSU crystals plus arhalofenate acid. (PDF 38585 kb) [file 13075_2018_1699_MOESM2_ESM.pdf]

Additional File 2

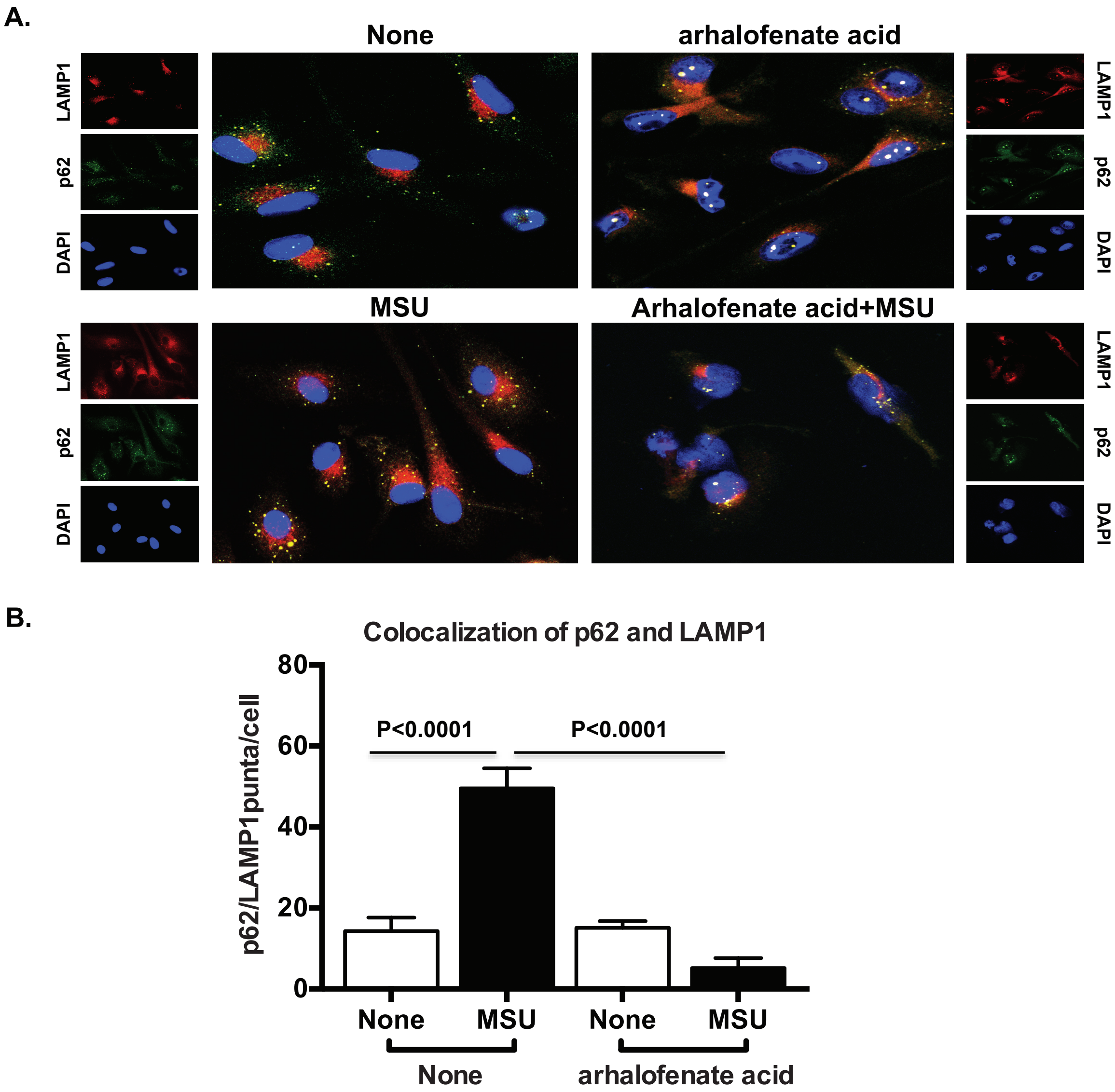

Additional file 2. Arhalofenate acid promoted autophagy flux. BMDMs were treated with arhalofenate acid (100 μM) for 1 hour before stimulated with MSU crystals (0.2 mg/ml) for 6 hours in RPMI containing 1% FBS. Immunofluorescence microscopy was carried out to visually identify p62 puncta (green) and lysosomes (LAMP1, red) and determine co-localization (yellow) of p62 and LAMP1 (A, 63X). The numbers of yellow punctae per cell were counted and presented in a graph (B). Data shown in A as representative of 3 individual experiments. Data in B as the mean±SD of 200 cells. P values represent comparisons between none and MSU crystals alone, or between MSU crystals alone and MSU crystals plus arhalofenate acid.
